# Multiple Intracranial Meningiomas: A Review of the Literature and a Case Report

**DOI:** 10.1155/2013/131962

**Published:** 2013-08-29

**Authors:** F. Koech, J. Orege, F. Ndiangui, B. Macharia, N. Mbaruku

**Affiliations:** ^1^Department of Neurosurgery, Moi University School of Medicine, P.O. Box 4606-30100, Eldoret 30100, Kenya; ^2^Department of Pathology, Moi University School of Medicine, P.O. Box 4606-30100, Eldoret 30100, Kenya; ^3^Department of Radiology, Moi University School of Medicine, P.O. Box 4606-30100, Eldoret 30100, Kenya; ^4^Department of Orthopedics, Moi University School of Medicine, P.O. Box 4606-30100, Eldoret 30100, Kenya

## Abstract

Multiple intracranial meningiomas are a condition where there is more than one meningioma in several intracranial locations in the same patient without signs of neurofibromatosis. Incidence varies from 1 to 10%. The prognosis of multiple intracranial meningioma does not differ from benign solitary meningiomas despite the multiplicity. However, the simultaneous occurrence of different grades of malignancy is observed in one-third of multiple meningiomas. Surgery remains the best option for treatment of symptomatic lesions. Our case review aims to present and discuss a 75-year-old female patient diagnosed with multiple intracranial meningiomas, describing their clinical, radiological, histological characteristics. It also highlights the fact that the patient had two tumours, underwent surgery, and so far has a good quality of life.

## 1. Introduction

Cushing and Eisenhardt [1] in 1938 were the first to coin the term meningioma. They came up with what closely resembles our contemporary understanding of the frequency of meningiomas by location. They defined multiple meningiomas as “at least two spatially separated meningiomas in a patient without signs of neurofibromatosis” [1]. The incidence of multiple meningiomas, defined by Cushing and Eisenhardt, is about 1 to 2 percent of all meningioma cases, and these results are comparable to those obtained by other authors [1].

## 2. Case Report

**Figure 1:**
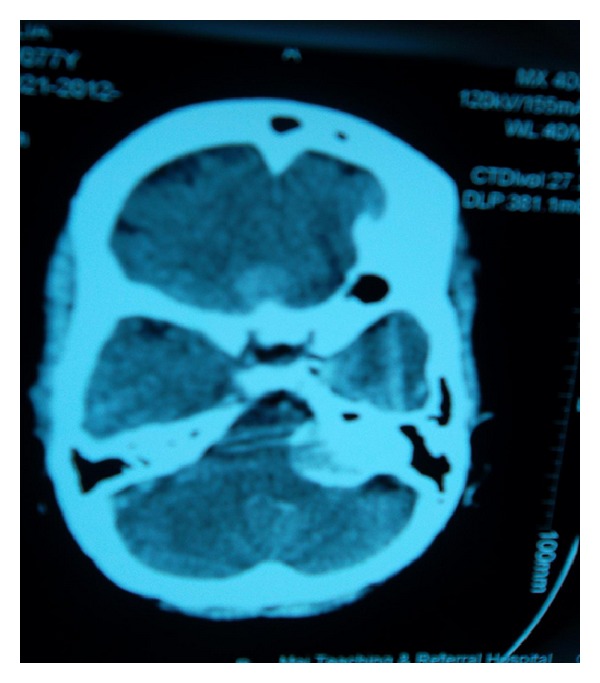
Axial CT scan of the head showing a meningioma in the left CP angle and another in the tuberculum sella.

**Figure 2:**
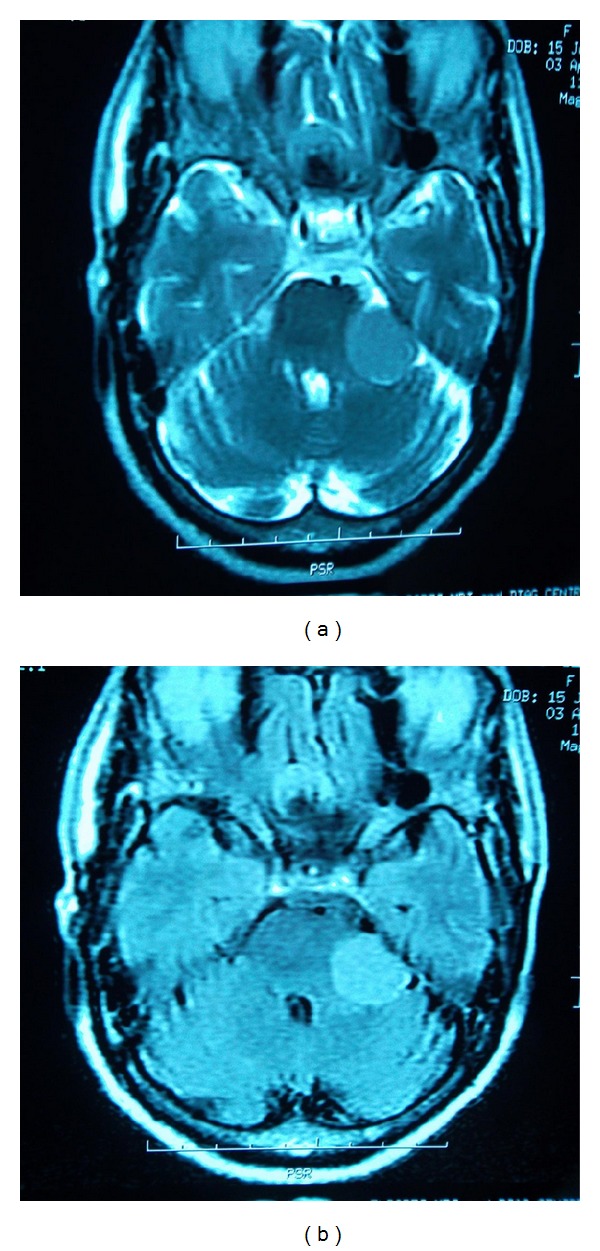
Axial T2W and FLAIR images showing a meningioma in the left CP angle and tuberculum sella (a) and (b).

**Figure 3:**
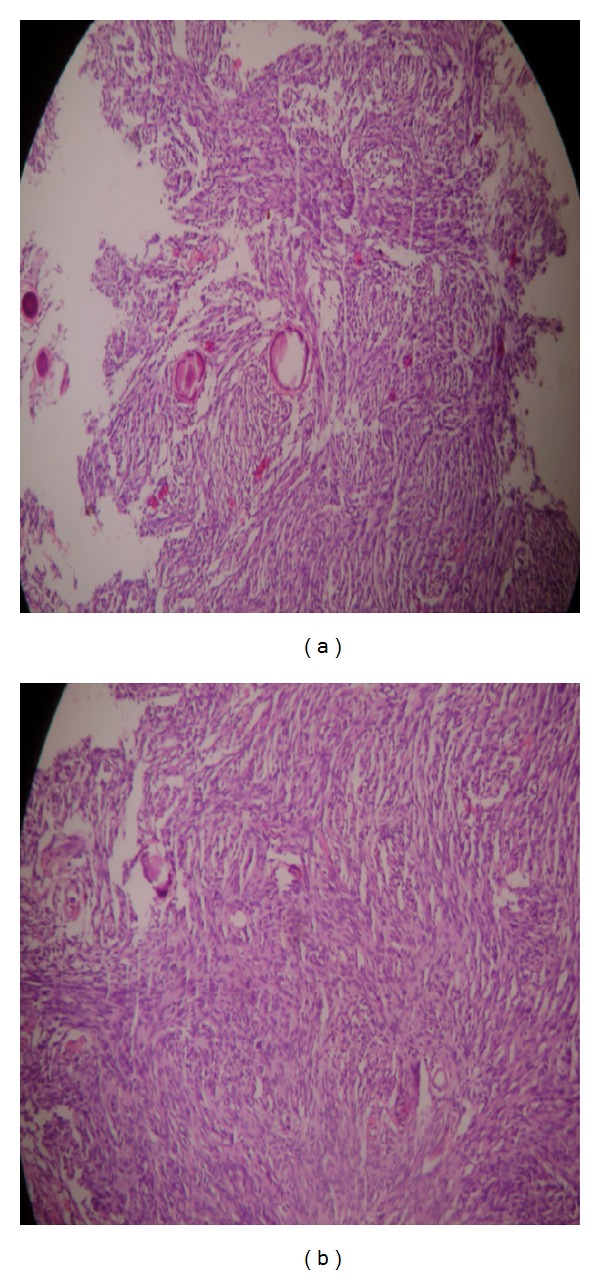
Histology results showing a fibroblastic meningioma (a) and (b).

A 75-year-old female patient presented with a six-month history of global headaches, loss of balance, and a change in facial sensation. She had no stigmata of neurofibromatosis. Central nervous system examination revealed reduced sensation on the left side, a GCS of 15/15, and all other cranial nerves were otherwise normal. Contrast enhanced CT scans (Figure 1) revealed two intracranial lesions, one in the left cerebellopontine angle (CPA) and another in the tuberculum sella area. T1W, T2W, and FLAIR magnetic resonance imaging scans (MRI) sequences showed hyperintense well circumscribed lesions in the left CPA and tuberculum sella (Figures 2(a) and 2(b)). The left infratentorial-CPA tumour showed significant mass effect. The MRI features of the two lesions were highly suggestive of multiple meningiomas. The patient was seen in the neurosurgery clinic and was scheduled for suboccipital craniotomy of the CPA tumour. This was removed achieving Simpsons Grade 2. The tuberculum sella lesion was not excised based on the age of the patient, small size of the tumour, and its lack of symptoms. Histological examination of the cerebellopontine lesion revealed a lesion composed of many sheets of fibroblastic cells separated by collagen bundles (Figures 3(a) and 3(b)). After surgery, she showed symptomatic improvement. Postoperatively, the patient improved remarkably with no headaches. The patient's first followup after surgery was unremarkable and is now scheduled for a second followup with a repeat MRI at 6 months and remains asymptomatic. 

## 3. Discussion 

Meningiomas are tumors originating from arachnoidal cells, granulations, stroma of the perivascular spaces, and in the choroid plexus, corresponding to 13 to 20% of all intracranial tumors [2]. The terminology multiple intracranial meningiomas should be used only when two or more meningiomas occur either simultaneously or sequentially in different locations [3]. The first quotations reveal an incidence of only 1 to 2%. With the introduction of CT and MRI, this incidence has increased [4]. This is attributable to better radiological diagnosis. With the advent of MRI, the incidences are reported to be even higher because MRI is more helpful than CT to detect the tumors particularly located in posterior fossa, skull base, and higher vertex area, especially when they are small. 

Kyoi et al. [5] encountered two patients with multiple meningiomas at their clinic. Locatelli et al. [6] reported ten cases of multiple meningiomas in a series of 227 intracranial meningiomas from 1977 to 1984. In this particular series, all the patients were female and underwent CT before operation. Domenicucci et al. [7] reported 14 cases of multiple intracranial meningiomas representing 1.1% of all meningiomas operated on at their hospital in the past 35 years. In their series, they noted that since the introduction of CT scanning, the frequency of these cases has risen from 0.58% to 4.5% in the authors' meningioma series. Gelabert-Gonzalez et al. [8] reported 13 cases of multiple intracranial meningiomas, consecutively operated on at their hospital between 1983 and 2003. In this particular series, all the patients were studied with CT and the last 10 with MRI, and all of the patients showed no manifestations of von Recklinghausen disease. Most of these cases of multiple meningiomas reported showed multiple lesions at the time of operation or after a few years of the initial operation.

One of the relevant etiological factors [9] that is important in the development of meningiomas is genetics. Studies have reported that the deletion of the chromosome 22 in patients with type 2 neurofibromatosis and in up to 50% of solitary meningiomas is connected with the appearance of multiple meningiomas. The second etiological factor is hormones. A number of papers show a higher frequency rate of meningiomas in women. One associated factor is the action of progesterone on progesterone receptors found in 80% of meningiomas, leading to an increase during the luteal phase of the menstrual cycle and during pregnancy [10]. In our case review, the patient was a postmenopausal female, and further endocrinological, genetic, and epidemiological studies will be needed to establish the pathogenesis of multiple meningiomas in this age group. 

The location of the tumours can be varied. There appears to be a tendency towards unilateral hemispheric localization, and the most common locations were the supratentorial convexity and the parasagittal falx, but multiple meningiomas occurring in the posterior fossa are very rare [11]. In our case review, one tumour was located at the cerebellopontine angle and another in the tuberculum sella. 

Histologically, multiple meningiomas do not differ from the solitary types [7]; however, the simultaneous occurrence of different grades of malignancy in the nodules is observed in one-third of multiple meningiomas [12]. The most common histological types reported in multiple meningiomas include psammomatous, fibroblastic, meningothelial, and transitional types [13]. In our case review, the histological type was a fibroblastic meningioma. 

The treatment and prognosis of multiple meningiomas do not differ from those of solitary benign tumours. Surgery is the treatment of choice for multiple meningiomas and depends on the following characteristics: symptomatic meningioma, asymptomatic meningioma greater than 3 cm in size and surgically accessible, and symptomatic expanding tumor [14, 15]. Each tumour should be approached individually, and the mere presence of multiple tumours does not justify their removal.

Since it is well documented that multiple intracranial meningiomas are histologically benign, the prognosis is good and may be the same as for solitary meningiomas. Some studies have shown no recurrence at follow-up evaluation [3, 7, 13]. 

Nakamura et al. [16] further highlight the natural history of asymptomatic meningiomas. In their study, the annual absolute growth rate ranges from 0.73 to 1.67 cm^3^ per year and the tumour doubling time ranges from 1.19 to 6.81 years. This growth varies with age, is minimal, and may thus be observed without surgical intervention unless specific symptoms develop.

 Additionally, an asymptomatic small meningioma should be followed up with MRI every 6 or 12 months if the patient is more than 65 years old. Surgery should be prescribed mainly for an expansive symptomatic tumour with cerebral edema. Our patient had two tumours of which only one was removed surgically, whereas the other was left to be followed up as it was small and asymptomatic.

## 4. Conclusion

Multiple meningiomas are rare. When they do occur they are more common in females. Their location can be varied, most commonly unihemispheric rarely in the posterior fossa. The histological subtypes are similar to the solitary types with the most common ones being psammomatous, fibroblastic, meningothelial, and transitional types. Definitive treatment is surgical removal of expansive tumours with cerebral edema and subsequent followup of small asymptomatic tumours.

## References

[1] Cushing  H, Eisenhardt L (1938). *Meningiomas: Their Classification, Regional Behaviour, Life History and Surgical End Result*.

[2] Russel DS, Rubinstein LJ (1977). *Pathology of Tumors of the Nervous System*.

[3] Turgut M, Palaoglus S, Ösmam OE, Gurçay G (1997). Multiples Meningiomas of the central nervous system without the stigmata of neurofibromatosis: clinical and therapeutic study. *Neurosurgical Review*.

[4] Butti G, Assietti R, Casalone R, Paoletti P (1989). Multiple Meningiomas: a clinical, surgical, and cytogenetic analysis. *Surgical Neurology*.

[5] Kyoi K, Yokoyama K, Hoshida T (1983). Mulitiple Meningioma. *No Shinkei Geka*.

[6] Locatelli D, Bottoni A, Uggetti C, Gozzoli L (1987). Multiple Meningiomas evaluated by computed tomography. *Neurochirurgia*.

[7] Domenicucci M, Santoro A, D’Osvaldo DH (1989). Multiple intracranial Meningiomas. *Journal of Neurosurgery*.

[8] Gelabert-Gonzalez M, Leira-Muino R, Fernandez-Villa JM (2003). Multiple intracranial Meningiomas. *Revista de Neurologia*.

[9] Black P, Morokoff A, Zauberman J, Claus E, Carroll R (2007). Meningiomas: science and surgery. *Clinical Neurosurgery*.

[10] Gruber T, Dare AO, Balos LL, Lele S, Fenstermaker RA (2004). Multiple Meningiomas arising during long-term therapy with the progesterone agonist megestrol acetate: case report. *Journal of Neurosurgery*.

[11] Kim TS, Park JK, Jung S (1997). Multiple intracranial Meningiomas. *Journal of Korean Neurosurgical*.

[12] Mocker K, Holland H, Ahnert P (2011). Multiple meningioma with different grades of malignancy: case report with genetic analysis applying single-nucleotide polymorphism array and classical cytogenetics. *Pathology Research and Practice*.

[13] Eljamel MSM, Foy PM (1989). Multiple Meningiomas and their relation to neurofibromatosis. *Surgical Neurology*.

[14] Salvati M, Caroli E, Ferrante L (2004). Zentrabl. *Neurochir*.

[15] Sheehy JP, Crockard HA (1983). Multiple Meningiomas: a longterm review. *Journal of Neurosurgery*.

[16] Nakamura M, Roser F, Michel J, Jacobs C, Samii M (2003). The natural history of incidental Meningiomas. *Neurosurg*.

